# Massive Nest-Box Supplementation Boosts Fecundity, Survival and Even Immigration without Altering Mating and Reproductive Behaviour in a Rapidly Recovered Bird Population

**DOI:** 10.1371/journal.pone.0036028

**Published:** 2012-04-24

**Authors:** Karine Berthier, Fabio Leippert, Luca Fumagalli, Raphaël Arlettaz

**Affiliations:** 1 Conservation Biology Division, Institute of Ecology and Evolution, University of Bern, Bern, Switzerland; 2 Swiss Ornithological Institute, Valais Field Station, Nature Centre, Salgesch, Switzerland; 3 Laboratory for Conservation Biology, Department of Ecology and Evolution, University of Lausanne, Lausanne, Switzerland; 4 School of Biological Sciences, University of Sydney, Sydney, New South Wales, Australia; Monash University, Australia

## Abstract

Habitat restoration measures may result in artificially high breeding density, for instance when nest-boxes saturate the environment, which can negatively impact species' demography. Potential risks include changes in mating and reproductive behaviour such as increased extra-pair paternity, conspecific brood parasitism, and polygyny. Under particular cicumstances, these mechanisms may disrupt reproduction, with populations dragged into an extinction vortex. With the use of nuclear microsatellite markers, we investigated the occurrence of these potentially negative effects in a recovered population of a rare secondary cavity-nesting farmland bird of Central Europe, the hoopoe (*Upupa epops*). High intensity farming in the study area has resulted in a total eradication of cavity trees, depriving hoopoes from breeding sites. An intensive nest-box campaign rectified this problem, resulting in a spectacular population recovery within a few years only. There was some concern, however, that the new, high artificially-induced breeding density might alter hoopoe mating and reproductive behaviour. As the species underwent a serious demographic bottleneck in the 1970–1990s, we also used the microsatellite markers to reconstitute the demo-genetic history of the population, looking in particular for signs of genetic erosion. We found i) a low occurrence of extra-pair paternity, polygyny and conspecific brood parasitism, ii) a high level of neutral genetic diversity (mean number of alleles and expected heterozygosity per locus: 13.8 and 83%, respectively) and, iii) evidence for genetic connectivity through recent immigration of individuals from well differentiated populations. The recent increase in breeding density did thus not induce so far any noticeable detrimental changes in mating and reproductive behaviour. The demographic bottleneck undergone by the population in the 1970s-1990s was furthermore not accompanied by any significant drop in neutral genetic diversity. Finally, genetic data converged with a concomitant demographic study to evidence that immigration strongly contributed to local population recovery.

## Introduction

Habitat degradation remains the main cause of decline and extinction of plant and animal species worldwide. It often affects some particular basic ecological requirements of species such as foraging grounds or breeding sites [1]. In birds, cavity-nesters have been particularly affected by the loss of breeding sites as a result of the intensification of sylviculture and agriculture [2]. The removal of snags and old trees by modern forestry and agriculture strongly limits the availability of suitable cavities for hole-nesting birds. The problem is further aggravated for those (secondary) cavity-nesters that are not able to excavate their own cavities and thus depend on pre-existing cavities. Traditional conservation measures for endangered cavity-nesting species rely on habitat conservation management that enhances the availability of natural suitable nesting sites in the landscape. However, habitat quality is often so dramatically reduced that supplementing natural cavities with nest-boxes is often necessary, at least during an initial transition period prior to renaturation of forests and hedges [3].

A threat for the long-term maintenance of populations artificially restored may yet emerge from the nest-box program itself. Artificial increase in population density may actually impact reproductive behaviours. Providing surplus nest-boxes to cavity-nesting birds may promote extra-pair paternity (EPP) [4], polygyny [5], and conspecific brood parasitism (CBP, i.e. egg dumping) [6], [7]. Through modifications of social interactions it can also negatively impact on individual fitness, i.e. compromise long-term population viability. In extreme cases, disruption of reproductive strategies may even drag populations into an extinction vortex [2].

The increasing use of molecular tools has shown that the rate of EPP is highly variable among and within species [8]. High breeding density, in interplay with other factors such as breeding synchrony, is a common explanation for high incidence of EPP [6], [9]. The main concern associated with nest-box supplementation programs is the potential dramatic increase of EPP due to an artificially generated high breeding density in species in which EPP is naturally infrequent [9]. In various cavity-nesting birds, supplementation of nest-boxes may also result in an increase of the frequency of polygyny (e.g. [5], [10]). In such cases, polygyny has generally been reported to affect the reproductive success of females with usually a higher cost for the second-mated female [11]. Again, reduced fitness of polygynously mated females may result from a lower level of assistance from male partners in feeding young and prevention of infanticide by conspecifics [12]. Common detrimental effects of CBP may be inefficient incubation of inflated clutches, increased risk of egg breakage, disturbance of the laying female by alien, parasitic females, if not nest abandonment [13], [14]. Effects of CBP on population dynamic stability are however not trivial depending on why and how parasitism occurs [15]. An important factor for a better understanding of the demographic consequences of CBP is whether or not it results from nest limitation. In this case, CBP is expected to increase the strength of density-dependence due to nest limitation and destabilize the dynamics [15]. Such a situation may lead to an increase of the frequency of CBP as observed for different box-nesting birds [4], [16], [17]. In some case, high frequency of CBP (i.e. >60%) may result in reduced individual reproductive success and lead to population instability and decline [2], [7], [13], [15], [18].

A nice example of a rapid population recovery after nest boxes installation is given by the hoopoe (*Upupa epops*, Linnaeus 1758) in Valais, Switzerland [3]. This migratory, secondary cavity-nesting bird has undergone dramatic declines across many parts of Europe. This species is now considered as one of the most endangered bird in Western and Central Europe [19]. Its conservation status varies according to the countries. In Switzerland, the spatial distribution of hoopoe has been reduced of 60% between the 1970s and 1990s and the species has been red-listed as highly endangered [19], [20]. The main Swiss population in Valais is isolated in the middle of the Alp Mountain with closest populations occurring hundreds of kilometres away in Italy, Spain, France and Germany. This population has been monitored since 1979 and a lack of suitable nesting cavities (e.g. woodpecker holes) on the intensively cultivated plain of the Rhone was identified as the principal factor limiting population development [20]. Fournier & Arlettaz [20] thus recommended the installation of nest-boxes in order to improve breeding conditions, which led to a spectacular and rapid demographic recovery and very high local densities [3]. A recent study implementing integrated population models from capture-mark-recapture data, data on fecundity and survey data showed that fecundity and juvenile survival were the main drivers of the hoopoe population dynamics since the installation of nestboxes in 1988; yet immigration had also a crucial role in the positive growth rate of the population [21].

Despite its demographic recovery, the future of the hoopoe population in Valais may still be of concern due to the potential alterations of the reproductive behaviour mentioned above. The natural rate of EPP in a low-density Spanish population of the hoopoe (7–8% of offspring [22]) was estimated moderate for a bird species [8], which may be explained by the high level of commitment of the male partner to female guarding and offspring feeding during the first weeks of the nestling period [23]. Yet, given the recent decline in breeding success in Valais [21], one can hypothesize a higher level of EPP in the more densely populated areas [24], [25], as female fitness may be affected by a reduction in food provisioning and nest defence from cuckolded males [26]. In addition, Martín-Vivaldi et al. [22] observed only a few cases in which an already paired male was intensively courting a second female, whereas such observations have been quite frequent in Valais (unpublished data). This suggests that polygyny may be a rare event in low-density hoopoe populations, but more frequent in high-density contexts. The absence of evidence for CBP in Spain [22] may similarly be explained by low breeding density. There is nevertheless always some risk for CBP considering that the hoopoe female often starts incubating only after laying of the third egg, which provides a time window for another female to dump eggs [23].

Because the Valais hoopoe population had undergone a strong reduction of its effective size until the late 1990s, when the restoration project started [3], it might theoretically still be prone to extinction due to a loss of genetic diversity through genetic drift and/or deleterious effects of inbreeding on reproduction and survival [27]. Effects of historical events impacting the genetic diversity, such as demographic bottlenecks, may persist for many generations [28] because the recovery of the genetic diversity then depends primarily on mutations and immigration. As mutation rates across the genome are very low, immigration is often the preponderant process to prevent the loss of genetic diversity, or restore it, in a bottlenecked population. Even though the effect of population bottlenecks on genetic diversity at neutral and functional loci may differ, assessing genetic variation at neutral markers, such as microsatellite loci, provides hints on the strength of the genetic drift, which will also impact the functional variation and then evolutionary processes within populations.

To assess the reproductive and genetic risks potentially at work in the Valais hoopoe population, we had first to develop and characterise new microsatellite markers for the species. We then performed parentage analyses in order to investigate the incidence and frequency of EPP, polygyny and CBP, while confronting our findings with those drawn from a low density Spanish population breeding in natural conditions (i.e. not supplemented by nest-boxes [22]). We also used these markers to evaluate whether i) the neutral genetic diversity has been negatively impacted by the past population decline and, ii) weather or not genetic data corroborate the importance of immigration in connecting the otherwise geographically isolated Valais population with others hoopoe populations [21].

## Materials and Methods

### Ethics Statement

All operations were carried out under licenses from the Swiss federal and cantonal authorities (reference: NS/yc - January 2004 - Département des finances, des institutions et de la sécurité and Service de la chasse, de la pêche et de la faune).

### Study area, population monitoring and sampling

The study was conducted on an area of ca 62 km^2^ on the plain of the Rhône in Central Valais (Southwestern Swiss Alps, 46°20′N, 7°40′E), where the main hoopoe population in Switzerland survives. The plain is used intensively by agriculture. Fruit tree plantations and vineyards are the main types of agricultural land use, whereas pastures, meadows and vegetable crops are scarce. Between 1998 and 2003, more than 700 nest boxes were installed in the area [3]. This led to a dramatic increase in population size until 2007 (see Figure 3 in [3]). Average yearly population growth rate between 1998 and 2007 was 32%. This translates into an almost sixfold increase in population size, from around 20 broods in the pre-nestbox period of 1998–2001, to 118 broods in 2007. About 90% of the asymptotic growth was reached in 2004, the year of the genetic sampling. After 2004, the increase was less rapid, with eventually a slight decrease in the last years, probably as a consequence of density-dependent regulation [21].

In 2004, 112 broods were recorded. Based on the maximal number of simultaneous broods, we estimated that at least 64 breeding pairs were present in the study area (1 pair per km^2^, but with local density up to 10 pairs km^2^ [3]). Genetic samples consisted of feathers collected from 254 juveniles belonging to 41 different broods. In addition, all adults visiting occupied nest boxes were captured with mist-nets and traps. Blood samples could thus be obtained from 76 adults (38 females and 38 males), considered as the social parents of the broods they visited. Feathers and blood samples were both stored at −20°C.

### Genetic diversity

Individual genotypes were determined at six microsatellite loci (Appendix S1). The following analyses were conducted on the adult genotype dataset. For each locus, the number of alleles and the observed (H_O_) and expected (H_E_) heterozygosities (unbiased estimates [29]) were computed using the program Genetix version 4.03 [30]. Genotypic linkage disequilibrium between all pairs of loci, and conformity to the Hardy–Weinberg equilibrium for each locus, were tested by exact tests using the Markov chain methods in Genepop software version 3.3 [31]. For each locus, deviation from Hardy–Weinberg equilibrium was quantified by the unbiased Wright inbreeding coefficient *F*_IS_, calculated according to Weir & Cockerham [32]. Corrections for multiple tests were performed using the false discovery rate approach (FDR) [33]. The presence of null alleles at each locus was investigated using the program Micro-checker and by checking for the presence of homozygous nulls in our genotypic data set [34]. The null allele frequency for each locus and averaged over loci was computed using the program Cervus 3.0.3 [35].

### Parentage analyses

Adult and juvenile genotypes were used to assess the incidence and frequency of EPP, polygyny and CBP. We conducted parent-pair analyses using the likelihood-based approach implemented in the software Cervus 3.0.3 [35], [36]. Parent-pair analysis is the optimal method of parentage testing when neither parent is known with 100% confidence, and when the objective is to assign both parents [35]. As sexes of candidate parents were known, Cervus assigned to each offspring the most likely mother, the most likely father, and the most likely parent pair. Parentage analyses were conducted on 254 juveniles from 41 different broods for which both the social mother (n = 38) and father (n = 38) had been sampled simultaneously. Simulation parameters in Cervus were: 10,000 cycles, 38 candidate mothers, 38 candidate fathers, 60% candidate mothers and fathers sampled (38 adult pairs sampled/64 estimated breeding pairs in 2004), genotypes available for 95% of loci and at least three typed loci required. By allowing for genotyping error, the software Cervus 3.0 allows to accommodate for the presence of null alleles that can be responsible for mismatches between putative parents and offspring and lead to false parentage exclusions [36]. We then assumed that 3% of loci were potentially mistyped (i.e. mean frequency of null allele per loci in our data set). A potential disadvantage to allow for typing error is however that non-parents that only mismatch at one or two loci may be falsely assigned [36]. To further minimize the effects of null alleles in parentage assignments, we have considered that a social parent can be excluded as being the biological one if i) mismatches occur at more than one locus and ii) mismatches are not due to the fact that juvenile and social parent are homozygotes for different alleles at one or more loci affected by null alleles, as recommended by Dakin & Avise [37].

### Immigration process

To evaluate recent immigration into the hoopoe population, we used the software GENECLASS 2.0 to detect first-generation migrants [38]. Likelihood computation was performed using the frequency method and the statistic *L_h_* [39], [40]. The probability for an individual to be an immigrant was assessed using the Monte Carlo resampling procedure of Paetkau et al. [40]. Only individuals assigned to the Valais population with a probability of less than 0.01 were considered as potential immigrants. The number of first-generation immigrants detected in our population sample consisting of 76 adults was used to evaluate the rate of immigration in the Valais population for the year 2004 (i.e. immigration rate = number of migrants/76 adults).

In a population experiencing a reduction of its effective size, rare alleles are lost quickly creating then a transient heterozygosity excess [41]. This particularity has been used by Cornuet & Luikart [42] to develop a method to detect recent genetic bottlenecks by computing the ΔH statistic which compares the expected heterozygosity (H_E_), as estimated within the population, with that expected on the basis of the observed number of alleles and sample size, in a similar population at mutation-drift equilibrium (H_eq_) (i.e. ΔH = H_E_−H_eq_). For an isolated population at mutation-drift equilibrium, the ΔH value should be equal to 0. A positive ΔH value (heterozygosity excess) would signal demographic reduction and a negative ΔH value (heterozygosity deficiency) would reveal demographic expansion. Using computer simulations and considering a stepping stone model, Pope et al. [43] examined the effects of various immigration rates on the behaviour of the ΔH statistics. They found that very low migration rates (e.g. <0.001) would result in negative ΔH values because immigrants from genetically differentiated populations bring new alleles in excess in respect to the heterozygosity. By contrast, a large amount of gene flow (i.e. high migration rates) between populations results in positive ΔH values because immigrants mostly bring alleles that are already present in the population, thus increasing heterozygosity but not the number of alleles. Based on the simulation results by Pope et al. [43], we used the ΔH statistics to investigate the role of immigration in the maintenance of the genetic diversity in the Valais hoopoe population. ΔH was computed for each locus and over all loci using the program Bottleneck [44]. We performed 10,000 iterations using the two-phase model of mutation (TPM) with a variance for TPM equal to 0.36 and a proportion of the stepwise-mutation model (SMM) in TPM equal to 0 [45]. Significant departure of the statistics ΔH from 0 was tested using a two-tailed Wilcoxon sign-rank test [44].

## Results

### Genetic diversity

The six microsatellite loci developed and characterized were all polymorphic (see Appendix S1). The number of alleles per locus observed within the adult population was comprised between 9 and 18 (Table 1). The expected and observed heterozygosities within the adult population ranged from 0.710 to 0.902, and from 0.625 to 0.927, respectively (Table 1). There was no significant linkage disequilibrium for all pairs of loci. After FDR correction, two loci (Upu935 and Upua2) showed a significant departure from Hardy-Weinberg equilibrium, corresponding to a heterozygosity deficit (Table 1). For these two loci, the excess of homozygotes was evenly distributed across most allele size classes, suggesting the presence of null alleles at these loci (Micro-checker [34]), which was also supported by the presence of genotype nulls at these loci where other loci were successfully amplified. Estimated frequencies of null alleles at the loci Upu935 and Upua2 were 0.07 and 0.147, respectively (Table 1).

**Table 1:** Characteristics of six microsatellite loci developed for *Upupa epops*.

Locus	Genbank accession	Primer sequences	Motif and Dye	Primer (µM)	A	Size range	*H*_O_	*H*_E_	HW	*F*_IS_	*f_NA_*	PE1	PE2
Upu 907	JQ742958	F: TGAATGAGCCTCCACTCTCC	(CA)_17_	0.2	13	116	0.89	0.85	0.370	−0.047	0.000	0.459	0.295
		R: CATTTCCCATACTGCCCG	FAM	0.2		142							
Upu 921	JQ742959	F: AGCTCTTGGTGAGGGCACTG	(GT)_12_	0.2	13	107	0.92	0.83	0.026	−0.112	0.000	0.495	0.325
		R: ACCCAGGAGTTCTCTCCAGG	HEX	0.2		121							
Upu 935	JQ742960	F: ACCCTGTACCCACACAAGTC	(TCTA)_12_	0.2	15	199	0.78	0.89	**0.010**	+0.129	0.072	0.348	0.210
		R: GAACAGCAGTTTGGACCTGC	NED	0.2		255							
Upu a2	JQ42961	F: TGTATCTGAGTCCATGGGGA	(CA)_21_	0.2	18	137	0.63	0.86	**0.000**	+0.268	0.147	0.445	0.284
		R: GCTGAGTGGCATGACCTGGA	NED	0.2		177							
Upu a3	JQ42962	F: CTGTTGTTACCACAGTGTC	(GT)_10_-GCCG-(GT)_6_	0.2	9	187	0.74	0.67	0.615	−0.103	0.000	0.702	0.521
		R: GCTCCTGACAAATACAGAAC	FAM	0.2		217							
Upu a7	JQ742963	F: GCCTTTCCACTTAGGCACCCG	(GT)_12_-GT-(GT)_3_	0.2	15	160	0.88	0.86	0.412	−0.018	0.000	0.447	0.285
		R: AGCAACGCCGCCACAATC	VIC	0.2		192							
All											0.036	0.011	0.001

Number of alleles, size range, observed heterozygosity (*H*_O_) and unbiased expected heterozygosity (*H*_E_) were estimated for each locus from 76 adult hoopoes population (Valais, Switzerland). HW indicates the probability associated with the rejection of the Hardy-Weinberg equilibrium; significant probabilities after FDR correction are indicated in bold. Null allele frequencies (*f*_NA_) and non-exclusion probabilities for the first (PE1) and second (PE2) parent were computed for each locus as well as averaged over loci using the program Cervus 3.0.3 (Marshall *et al.* 1998).

### Parentage analyses

**Figure 1:**
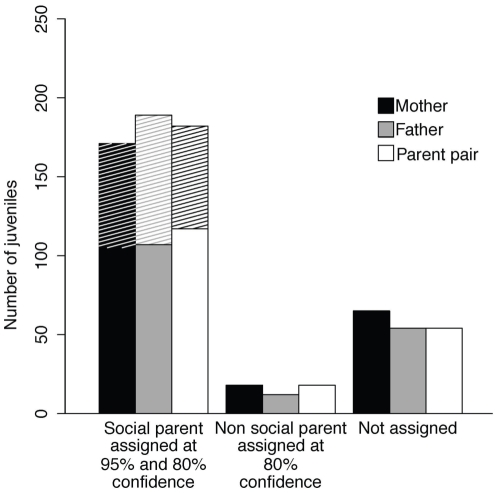
Number of maternities, paternities and parental pairs assignments. Numbers of maternities (black), paternities (grey) and assignments to parental pairs (empty) are given for i) the social parents (social mother, social father, and social parental pair) with 80% (total bars) and 95% (non-hatched bars only) confidence, ii) non social parents (mother, father and parental pairs) with 80% confidence; and iii) number of juveniles for which maternity, paternity and parental pairs could not be properly assigned with 80% confidence.

The probability combined over all loci of not excluding a single unrelated candidate parent was relatively small: 0.011 and 0.001 for the first and second parent, respectively, providing an adequate confidence level when assigning paternity with our 6 selected microsatellites (Table 1). Results of parentage analyses using Cervus (Figure 1 and Table S1) showed that paternity alone was assigned to the social father with at least 80% confidence for 189 out of 254 juveniles (107 were assigned with 95% confidence). For 12 out of the 65 remaining juveniles, paternity was assigned to a male other than the social one with at least 80% confidence. These 12 juveniles belonged to 7 different broods and among them the social father was the second most likely father in 9 cases. Among the 53 remaining juveniles, the social father was the most likely biological father in 46 cases, but was assigned with less than 80% confidence (Table S1). Maternity alone was assigned to the social mother with at least 80% confidence for 171 out of 254 juveniles (105 were assigned with 95% confidence). For 18 out of the 83 remaining juveniles, the maternity was assigned to a mother other than the social one with at least 80% confidence. These 18 juveniles belonged to 13 different broods and among them the social mother was the second most likely mother in 14 cases. Among the 65 last juveniles, the social mother was the most likely biological mother in 50 cases, although maternity was here assigned with less than 80% (Table S1). Parent pairs including the two social parents were assigned with at least 80% confidence for 182 out of 254 juveniles (117 were assigned with 95% confidence). In 18 cases the parent pair assigned included at least one non-social parent. For the 54 remaining juveniles, none of the parent pairs were assigned with a least 80% confidence (Figure 1). When the maternity or paternity was not assigned to the social father (65 cases) or social mother (83 cases), we checked visually the genotypes of all individuals involved in the trio (i.e. assigned mother, assigned father, juvenile) as well as the genotypes of the social mother and social father. In 5 out of the 30 cases, where the paternity (12 cases) or maternity (18 cases) was assigned with at least 80% confidence to a parent other than the social one, the latter showed no mismatch between its genotype and the juvenile genotype. In the 25 other cases, visual inspection revealed inconsistencies between the genotypes of the juvenile and social parent, mother or father, which all arose from the loci affected by null alleles, i.e. Upu935 and Upua2. In all cases for which the paternity and maternity were not assigned with at least 80% confidence (53 and 65 cases, respectively) we observed at least one mismatch between juvenile and adult genotypes at the loci affected by null alleles. In a few cases, the genotype of the juvenile and/or social parents was missing at one of the two loci affected by null alleles, which could be due to homozygote null allele genotypes. Other mismatches occurred because the social parent and the juvenile were apparently homozygous for different alleles, which is likely to be due to null alleles.

We also found in our data that only one female reproduced with two different males, while one of the latter also reproduced a second time with a different female.

### Immigration process

Detection of first-generation migrants using GENECLASS resulted in four individuals, three males and one female, being inferred to enter the population as immigrants in 2004 (probability ranged from *P*<0.0001 to *P* = 0.009). These four individuals were all carrying low frequency (i.e. <3%) or novel alleles at least for two of the six microsatellite loci analysed. Immigration rate estimated by dividing the number of first-generation migrants (i.e. 4) by the number of adults captured in 2004 and included in the genetic data set (i.e. 76) was 0.053.

The mean value of the statistic ΔH, computed over the six loci was −0.033, which was significantly different from the ΔH value expected under mutation-drift equilibrium (i.e. ΔH = 0, Wilcoxon sign-rank test P = 0.046). Continuous immigration at a rate of 0.05 in a population of about 100 individuals is expected to produce high positive ΔH values (i.e. ∼0.03) [43]. The negative ΔH value found for the Valais hoopoe population could result from the recent and sudden arrival of a few immigrants carrying rare or even novel alleles for this population, as suggested when looking at the genotypes of the individuals detected as first-generation migrants in the hoopoe population in 2004.

## Discussion

Microsatellite data and parentage analyses revealed that juveniles were mainly assigned to the social father and mother (74% and 67%, respectively). The number of juveniles for which the paternity or the maternity was not assigned with at least 80% confidence (21% and 26%, respectively) or assigned to a male other than the social one (5%) or a female other than the social one (7%) should provide estimates of the potential frequency of EPP (26%) and CBP (33%) in the Valais hoopoe populations. However, following the recommendations of Dakin & Avise [37], we did not exclude that the social mother or the social father can be the biological parent when only one mismatch occurred, due to apparently homozygous or homozygous null genotypes, at a locus affected by null alleles. We suggest therefore that most of the cases where the maternity or paternity was not assigned with at least 80% confidence to the social parent might reflect some lack of statistical power because parentage assignments were based on 6 loci among which two are affected by the presence of null alleles. Moreover, there is no obvious reason for the social father or mother to be listed as one of the two most likely parents if these cases represent real EPP and CBP events. In our data, we found 10 and 19 cases for which the social father or mother is not one of the two most likely parents, respectively. This would represent a rate of EPP and CBP of about 4 and 7%, respectively in the Valais hoopoe population. Although breeding density was about five times higher in 2004 compared to the situation prior to nest box installation [3], these low levels of EPP and CBP are consistent with previous observations made within a natural, low-density hoopoe population (i.e. no case of CBP and a moderate rate of EPP of 7–8% in a Spanish study [20]). We also did not find any clear evidence for a high level of polygyny, as only one male was found to be the social and biological father of two broods held by two different females.

Hoopoe's species-specific behavioural traits as well as the locally implemented management measures may both explain these results. Although hoopoes are not very territorial, with overlapping home ranges, they aggressively defend the surroundings of their nest site against conspecifics, which probably reduces opportunities for both egg dumping and extra-pair paternity. Finally, despite high local density in core areas, nest sites in Valais may not be a limiting factor, with more than 700 nest boxes available to fewer than 100 pairs on an area of about 62 km^2^ and local densities of up to 10 pairs/km^2^. This may contribute to limit the occurrence of EPP and CBP events.

Despite its historical reduction in size the local population showed, in 2004, a substantial genetic diversity (H_E_ averaged over loci = 0.83; average number of alleles per locus = 14), which is among the highest reported in birds [46]. Moreover, genetic data showed evidence for recent immigration events into the population (i.e. four putative immigrants detected in the 2004 sample). The computation of the ΔH statistics brings further insights on the immigration process. In the study population, mean ΔH value over 6 loci, population size and immigration rate estimates were about −0.033, 128 and 0.053, respectively. According to Pope et al. [43], an immigration rate of about 0.05 in a population of 100 individuals would produce high positive ΔH values about ∼0.03 at mutation-drift-migration equilibrium (i.e. mean ΔH value over 7 loci, averaged over 1000 iterations for 10 populations). The discrepancy between our results and the simulations by Pope et al. [43] may lie in a very recent and sudden immigration into the Valais population, contrary to what is envisioned in Pope's model (i.e. continuous immigration) from genetically differentiated populations. Indeed, the four putative immigrants detected within the Valais hoopoe population in 2004 were all carrying rare or novel alleles mimicking a genetic signal for demographic expansion in a closed population when applying the heterozygosity test developed by Cornuet & Luikart [42]. These findings suggest that the Valais hoopoe population has been receiving immigrants from genetically well-differentiated populations during the demographic recovery process.

These genetic findings are in line with recent mark-recapture demographic analyses of the same hoopoe population [21]. The very rapid local demographic recovery was clearly caused by a new, unlimiting offer of suitable breeding sites (nest boxes) on the plain, which was literally void of breeding opportunities before the nest-box campaign. This sudden massive availability of nesting sites elicited an immediate and widespread move into the plain of the population that was previously breeding on the foothill slopes which once offered the only available breeding sites [3]. The installation of the breeding hoopoes on the plain, close to their most profitable prey, mole crickets, resulted in much shorter provisioning flights, i.e. less energy-demanding prey transportation costs for the parents [20], [23]. Results from integrated population models using capture-mark-recapture data, data on fecundity and survey data showed that the restoration of the breeding sites on the plain translated into higher fecundity and juvenile survival (i.e. higher recruitment), which were the two main drivers of population growth [21]. Yet, these demographic analyses also indicated that immigration contributed importantly to the positive growth rate, with an average annual immigration rate as high as 28% (concerning mostly one-year immigrants, i.e. natal dispersal) [21]. The numerous nest boxes are thus likely to have attracted and retained individuals that initially just stopped over in Valais, when crossing this region during their spring migration, especially due to the central position of Switzerland along the migratory routes of this long-distance, trans-saharian migrant [47].

### Conclusion

Given that behavioural dynamics can sometimes exert more negative effects on population dynamics than mere extrinsic factors, such as weather or habitat quality, attention must be paid to relevant behavioral interactions in species conservation programs [2]. Molecular approaches using genetic markers have greatly contributed to decipher behavioural patterns in animal populations (reviewed in [48]). Using microsatellite markers, this study evaluated the risks, potentially elicited by an artificially increased breeding density, for the mating and reproductive behaviour in a rescued hoopoe population. However, we could not detect any such detrimental changes suggesting then *a posteriori* the innocuousness of the massive nest box campaign implemented for rescuing this threatened population. The hoopoe population showed substantial genetic diversity that further suggested it has recently been genetically connected to other differentiated populations despite its apparent topographic isolation in a deep valley in the middle of the Alps, with the next populations situated hundreds of km away. Hence, genetic data converged with demographic data [21] in demonstrating connectivity [49] and evidencing the important role played by immigration in the demographic recovery process of this once threatened, now rescued bird population.

## Supporting Information

Appendix S1 **Microsatellite characterisation and genotyping.**
(DOC)

Table S1 **Details of the results of the parentage assignment analysis with a 80% confidence threshold.**
(DOC)
