# Supplementary material for: Massive Nest-Box Supplementation Boosts Fecundity, Survival and Even Immigration without Altering Mating and Reproductive Behaviour in a Rapidly Recovered Bird Population
Source: PLoS One. 2012 Apr 24;7(4):e36028. doi: 10.1371/journal.pone.0036028 (PMC3335799; doi:10.1371/journal.pone.0036028)
Supplement: Appendix S1 — Microsatellite characterisation and genotyping. (DOC) [file pone.0036028.s001.doc]

**Appendix S1**

Genomic DNA was extracted from feather and blood samples after digestion with 20 and 40 µl of proteinase K, respectively, and 500 µl of TNES-UREA buffer (10mM Trizma base, 0.3M NaCl, 1% SDS, 10 mM EDTA, 4M Urea). The mixture was incubated at 55°C and regularly shaken during at least three hours. After incubation, 400 µl of the digestion product was mixed with 20 µl of Magnesil Solution (Promega). DNA was isolated using the MagnaBot 96 Magnetic Separation Device (Promega). The Magnesil pellet was washed twice using 90% ethanol and 70% ethanol, successively.

Individual genotypes were determined at six microsatellite loci developed by a private company (Genetic Identification Services, Chatworth, USA) using an enriched library protocol. Amplification was carried out in one single multiplex reaction mixture of 10 µl containing 1 x QIAGEN Multiplex PCR Master Mix (including *Taq*, dNTPs and 3 mM of MgCl_2_ as final concentration), 0.2 µM of each primer, and 20 ng of genomic DNA. The multiplex PCR was performed on a GeneAmp thermocycler 9700 (Applied Biosystem) as follows: initial activation step at 95°C for 15 min followed by 30 cycles with denaturation at 94°C for 30 s, annealing at 57°C for 90 s and extension at 72°C for 90 s. The PCR products were diluted 3-fold, and 1 µL of the diluted-PCRs was mixed with 0.25 µL of Genescan 500 LIZ standard and 9.75 µL of Hidi Formamid (Applied Biosystem). The samples were separated and visualized on an ABI 3100 automated sequencer, and analyzed using GeneMapper v. 3.0 (Applied Biosystem).
