# Supplementary material for: Massive Nest-Box Supplementation Boosts Fecundity, Survival and Even Immigration without Altering Mating and Reproductive Behaviour in a Rapidly Recovered Bird Population
Source: PLoS One. 2012 Apr 24;7(4):e36028. doi: 10.1371/journal.pone.0036028 (PMC3335799; doi:10.1371/journal.pone.0036028)
Supplement: Table S1 — Details of the results of the parentage assignment analysis with a 80% confidence threshold. (DOC) [file pone.0036028.s002.doc]

**Table S1**

| **Brood ID** | **Brood size** | **Social father** | **Social mother** | **Social pair** | **Non social father** | **Non social mother** | **Non social pair** |
| --- | --- | --- | --- | --- | --- | --- | --- |
| 1 | 7 | 7 | 7 | 7 | / | / | / |
| 2 | 6 | 2 | 6 | 6 | 0 | / | / |
| 3 | 6 | 3 | 5 | 5 | 0 | 0 | 0 |
| 4 | 5 | 2 | 1 | 1 | 0 | 0 | 0 |
| 5 | 3 | 2 | 2 | 2 | 0 | 1 | 1 |
| 6 | 9 | 7 | 8 | 7 | 1 | 0 | 1 |
| 7 | 7 | 5 | 6 | 6 | 0 | 1 | 0 |
| 8 | 7 | 5 | 4 | 5 | 0 | 0 | 0 |
| 9 | 7 | 7 | 7 | 7 | / | / | / |
| 10 | 8 | 7 | 8 | 7 | 0 | / | 1 |
| 11 | 7 | 4 | 6 | 3 | 0 | 0 | 0 |
| 12 | 8 | 8 | 4 | 6 | / | 0 | 2 |
| 13 | 7 | 5 | 0 | 0 | 1 | 0 | 0 |
| 14 | 5 | 4 | 3 | 5 | 0 | 0 | 0 |
| 15 | 6 | 2 | 4 | 4 | 0 | 1 | 0 |
| 16 | 6 | 3 | 0 | 0 | 0 | 4 | 1 |
| 17 | 6 | 5 | 3 | 3 | 1 | 1 | 1 |
| 18 | 2 | 1 | 1 | 0 | 0 | 0 | 0 |
| 19 | 5 | 2 | 5 | 4 | 0 | / | 0 |
| 20 | 5 | 5 | 3 | 5 | / | 0 | / |
| 21 | 6 | 5 | 2 | 1 | 0 | 0 | 2 |
| 22 | 5 | 4 | 5 | 5 | 0 | / | / |
| 23 | 5 | 1 | 5 | 1 | 1 | / | 1 |
| 24 | 9 | 9 | 7 | 9 | / | 2 | / |
| 25 | 7 | 6 | 5 | 7 | 0 | 1 | / |
| 26 | 5 | 4 | 5 | 4 | 0 | / | 0 |
| 27 | 7 | 6 | 3 | 7 | 0 | 1 | / |
| 28 | 4 | 4 | 2 | 4 | / | 2 | / |
| 29 | 5 | 0 | 3 | 4 | 4 | 0 | 1 |
| 30 | 7 | 3 | 4 | 3 | 0 | 0 | 0 |
| 31 | 6 | 5 | 6 | 6 | 0 | / | / |
| 32 | 4 | 4 | 3 | 4 | / | 0 | / |
| 33 | 7 | 6 | 4 | 5 | 0 | 0 | 1 |
| 34 | 7 | 7 | 3 | 5 | / | 1 | 0 |
| 35 | 6 | 6 | 2 | 2 | / | 1 | 3 |
| 36 | 7 | 4 | 7 | 7 | 0 | / | / |
| 37 | 7 | 7 | 6 | 7 | / | 1 | / |
| 38 | 9 | 6 | 5 | 4 | 2 | 0 | 1 |
| 39 | 4 | 4 | 3 | 4 | / | 0 | / |
| 40 | 7 | 4 | 1 | 2 | 2 | 1 | 2 |
| 41 | 8 | 8 | 7 | 8 | / | 0 | / |
| Total | 254 | 189 | 171 | 182 | 12 | 18 | 18 |
